# High-confinement alumina waveguides with sub-dB/cm propagation losses at 450 nm

**DOI:** 10.1038/s41598-023-46877-4

**Published:** 2023-11-14

**Authors:** Elissa McKay, Natale G. Pruiti, Stuart May, Marc Sorel

**Affiliations:** 1 University of Glasgow, Glasgow, UK; 2 Istituto di Tecnologie della Comunicazione, dell’Informazione e della Percezione, Pisa, Italy

**Keywords:** Integrated optics, Optical materials, Materials for optics, Optical materials and structures

## Abstract

Amorphous alumina is highly transparent across the visible spectrum, making it a promising candidate for low-loss waveguiding at short wavelengths. However, previous alumina waveguide demonstrations in the visible region have focused on low- to moderate-confinement waveguides, where the diffuse mode reduces the design flexibility and integration density of photonic integrated circuits. Here, we have developed a high-quality etch mask and a highly selective BCl_3_ plasma etch, allowing etching of amorphous alumina waveguides up to 800 nm thick. Using this process, we have fabricated waveguides using an alumina film grown by atomic layer deposition (ALD) which are the lowest-loss high-confinement waveguides for blue light to date: we achieve single-mode propagation losses of 0.8 dB/cm at a propagation wavelength of 450 nm.

## Introduction

Low-loss waveguides for the visible region facilitate a range of applications in nonlinear optics, quantum optics^1^, and optical timing^2^. Whilst several mature, high-quality material platforms for the infrared (IR) region exist, the development of similarly robust and flexible platforms for the visible region remains a challenge. Propagation losses are considerably higher in the visible region than in the IR, since both absorption and scattering losses increase substantially as wavelength shortens. Band edge absorption places a fundamental limit on the transparency window of materials, prohibiting the use of well-established silicon-on-insulator (SOI) and most III-V platforms in the visible region and limiting the utility of silicon nitride (SiN) in the blue and ultraviolet (UV)^3,4^. Bulk scattering losses arise primarily from Rayleigh scattering, which varies with $1/\lambda^{4}$, whilst interface scattering varies with approximately $1/\lambda^{3}$^4,5^; consequently, compared with IR waveguiding, visible waveguiding places far higher demands on material and fabrication quality.

Propagation losses can be reduced by designing low-confinement waveguides, where much of the mode volume travels in the cladding rather than the core. This minimises losses associated with the core material (intrinsic and extrinsic absorption losses, as well as bulk scattering) and with fabrication (typically, thinner layers facilitate high-quality deposition and waveguide definition processes). However, thicker, higher-confinement waveguides widen process windows for heterogeneous and hybrid source integration, and allow for more design flexibility. Applications in nonlinear optics may require waveguides without an upper cladding layer in order to access the anomalous dispersion regime^6,7^. Omission of the upper cladding presents further challenges for fabrication processes: the higher refractive index difference between the core and (air) cladding produces substantially higher scattering losses^5^, placing even higher demands on lithography and etch quality. Consequently, there are very few demonstrations of unclad waveguides in the visible region.

Producing high-confinement and low-loss waveguides—especially unclad waveguides—requires a material with little intrinsic and extrinsic absorption in the target region, high homogeneity (to minimise bulk scattering), and a combination of mask and etch which is sufficiently selective and smooth. Amorphous alumina has emerged as a frontrunner in the field of blue and UV optics, the only material platform for which losses below 1 dB/cm have been realised below a wavelength of 500 nm for waveguides with a core thickness of at least 100 nm^8^. Additionally, there are several demonstrations of low-loss operation into the UV^8,9^. Whilst bandgap values as low as 200 nm have been reported for amorphous alumina films^10^, the optical quality of amorphous alumina films is highly dependent on deposition parameters and achieving consistent results can be challenging^11,12^.

Atomic layer deposition (ALD) offers a pathway to more consistent optical-quality film depositions: whilst careful control and optimisation of conditions is required^13^, ALD processes have demonstrated high run-to-run consistency, even across different deposition tools^14,15^ and substrate profiles^16^. The deposition method uses alternating, self-limiting half-reactions to deposit films which are inherently conformal and homogenous (minimising scattering losses) and high-purity (minimising extrinsic absorption losses). However, cyclic deposition of sub-monolayers, with chamber purges between each deposition step, is an extremely slow process, which may limit the viability of depositing thick ALD films. Prior work on optical-quality alumina films has almost exclusively used thermal ALD processes, where water vapour is used as the oxidant^8,9,17^; this reactant is notoriously difficult to purge from the reaction chamber^14^, and water vapour purging accounts for a substantial proportion of the cycle time. Whilst oxygen plasma can be used as an alternative oxidising agent^18^ offering shorter cycle times and higher growth per cycle (GPC)^14^, films grown using this plasma ALD process have not previously been assessed for their suitability for visible waveguiding.

Furthermore, defining structures in alumina films is challenging. Relative to standard mask materials, alumina has a slow etch rate in typical halide chemistries^19^. For infrared applications, where sidewall roughness is less crucial, the use of thick photoresist masks is well-established, reducing the selectivity requirement^19,20^. However, no results in the visible region have been reported for alumina waveguides thicker than 120 nm^21^, or with a confinement factor higher than 0.4^8^. Additionally, there are few reported loss results for alumina waveguides—or any waveguides optimised for the visible regime—without an upper cladding, where the high index contrast between the core and the surrounding air produces higher levels of interface scattering^5^. Consequently, whilst amorphous alumina appears to be a promising candidate for blue and UV waveguiding, the existing literature does not provide a robust demonstration of its functionality for photonic integrated circuit (PIC) applications. Here, we describe optimised deposition by plasma ALD, lithography, and etch processes for the fabrication of amorphous alumina waveguides, and demonstrate low propagation losses in the visible region with a confinement factor above 0.7 for both cladded and unclad geometries.

## Fabrication

A full fabrication process flow is provided in the “Methods” section; here, we describe the process development work which was central to producing high-confinement waveguides with low optical losses.

### Film deposition

**Figure 1:**
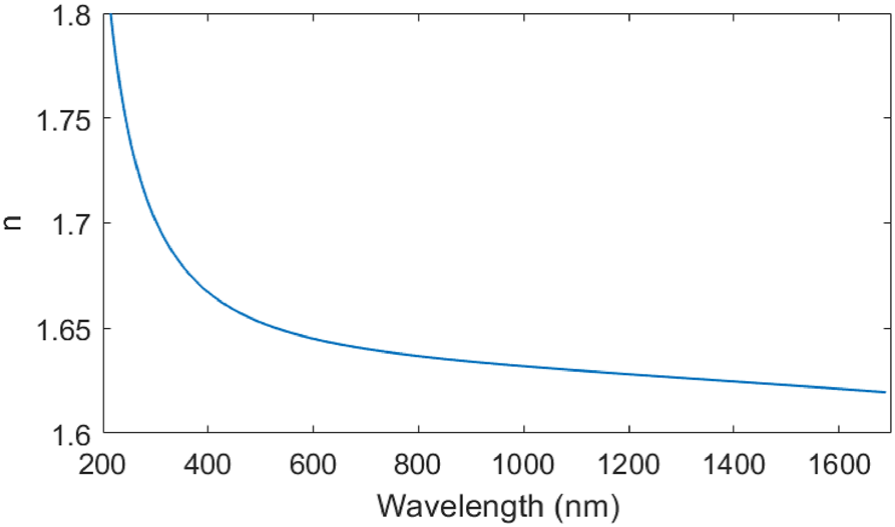
Chromatic dispersion of amorphous alumina film deposited using plasma ALD process. Dispersion is derived from multiple angle ellipsometry and fitting to a Tauc-Lorentz model^22^.

The films were deposited by ALD, using oxygen plasma oxidation to increase the deposition rate. Compared to the more standard thermal process with trimethylaluminium (TMA) and water reactants, the oxidation plasma process reduces the deposition time for a 400 nm-thick layer by over 60%, from 13.5 to 5.2 h. Eliminating the need to purge water vapour from the chamber reduces purging time by 6 s, and reduces the time for a single cycle from 12.17 to 6.02 s. However, the plasma process also has a high GPC, depositing 1.3 Å per cycle compared to 1.0 Å per cycle using the thermal process. This higher GPC arises from more efficient oxidation of the aluminium centres^18^. The deposited films have a refractive index of 1.66 at 450 nm (Fig. 1) and a topside roughness of 0.69 ± 0.03 nm. Ellipsometry and atomic force microscopy procedures are described in the “Methods” section.

### Lithography

**Figure 2:**
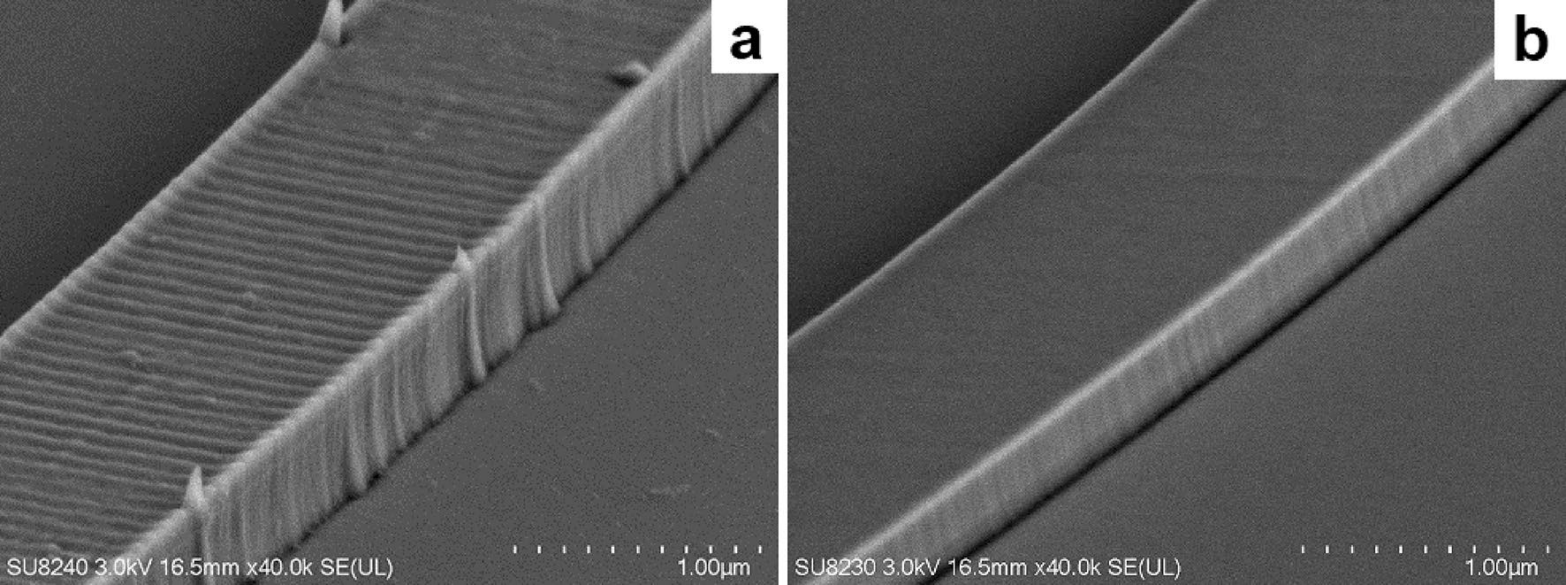
Scanning electron micrographs showing etched alumina waveguides exposed using standard and optimised lithography conditions. The optimised conditions produce a substantially smoother waveguide. (**a**) Standard lithography conditions. (**b**) Optimised lithography conditions.

We use hydrogen silsesquioxane (HSQ) as an etch mask, a negative resist which produces a silica-like structure upon electron-beam lithography (EBL) exposure. With optimisation, HSQ forms extremely smooth, vertical masks^23^; however, the lithography conditions are critical to producing good results. Figure 2 shows two curved waveguides after etch, one exposed using standard process conditions and the other after lithography optimisation: the difference in lithographic roughness is evident. This result required several process modifications:

- Multipass exposure, splitting the exposure dose over 4 passes to reduce sidewall roughness and mitigate field stitching errors^24,25^
- A small beam step size (1 nm) to minimise the effect of shot filling patterns, especially at waveguide edges
- An overlap of ${1}\,{\upmu }\text {m}$ at the edge of exposure fields, to mitigate stitching
- A beam height offset to eliminate field scaling errors caused by substrate and resist transparency to the height gauge laser

### Etching

**Figure 3:**
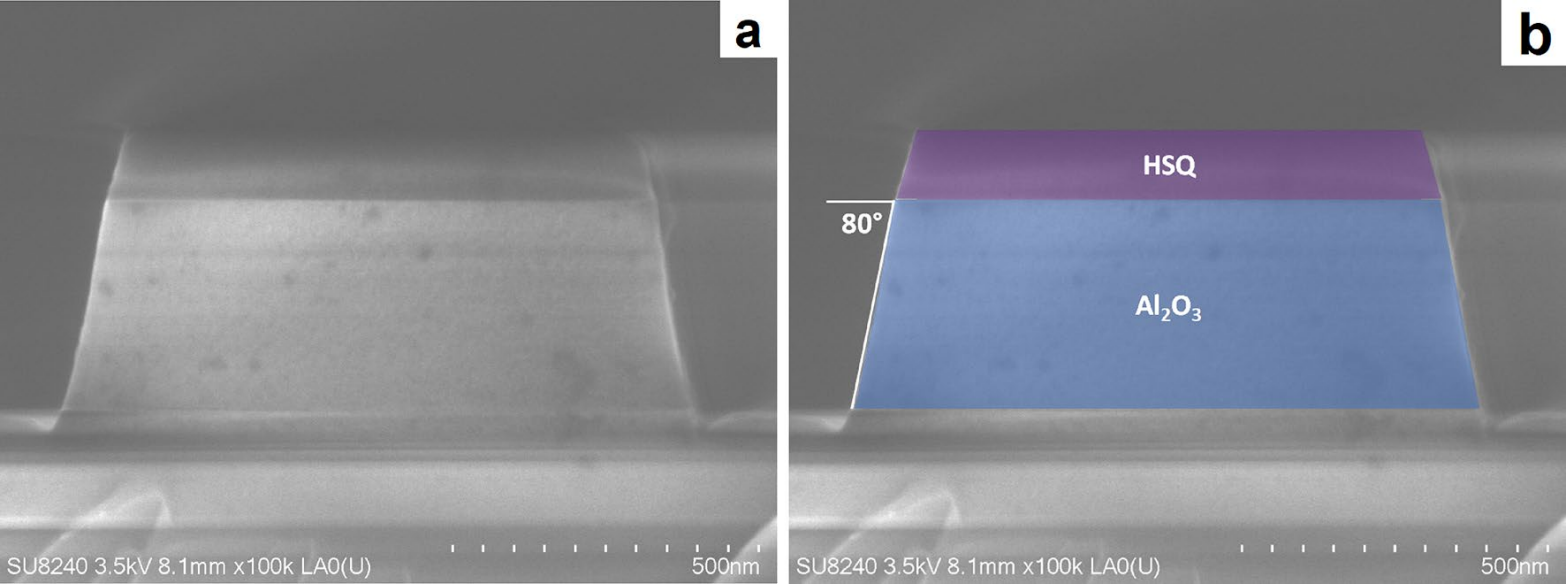
Scanning electron micrograph showing cross-section of a 400 nm-thick, 850 nm-wide alumina waveguide after etching. Sidewall angle is $\sim 80^{\circ }$. About 100 nm of HSQ remains on the top of the waveguide. (**a**) shows the original micrograph; (**b**) presents a false-colour image for enhanced clarity.

To define the optical waveguides, we use an ICP-RIE (inductively coupled plasma reactive-ion etching) process with BCl$_{3}$ gas. Alumina is readily etched by halide species, and the etch by-products are volatile. ICP-RIE etching allows for independent control of platen and ICP coil powers, and therefore provides precise control of ion etch rate. Etch selectivity tests indicate that a low negative DC bias improves etch selectivity, likely by emphasising the chemical component of the etch over the physical component. Lower DC bias values produce a lower mean free path for ionic species; a higher-bias etch may cause more damage even if increased etch rate reduces the overall time the sample is exposed to the plasma^26^. Increasing ICP power increases ion density, and therefore increases etch rate of both species. Increasing platen power increases etch rate for both mask and substrate, but also increases DC bias. Therefore, the lowest feasible platen power (15 W) and highest ICP coil power (1400 W) provide maximum selectivity (0.8); however, lower-power etches produce smoother sidewalls. Based on these considerations, we used an optimised etch with an ICP power of 600 W and a platen power of 25 W, which produces a sidewall angle of $80^{\circ }$ (Fig. 3) with very smooth sidewalls (Fig. 2) and a selectivity of 0.65. The HSQ mask cannot be removed following etching without negatively affecting the quality of the waveguide sidewalls; however, ellipsometry of exposed HSQ did not demonstrate absorption within the range of the tool (200–1700 nm) so we do not expect any increase in propagation loss due to absorption in the HSQ mask.

## Results

**Figure 4:**
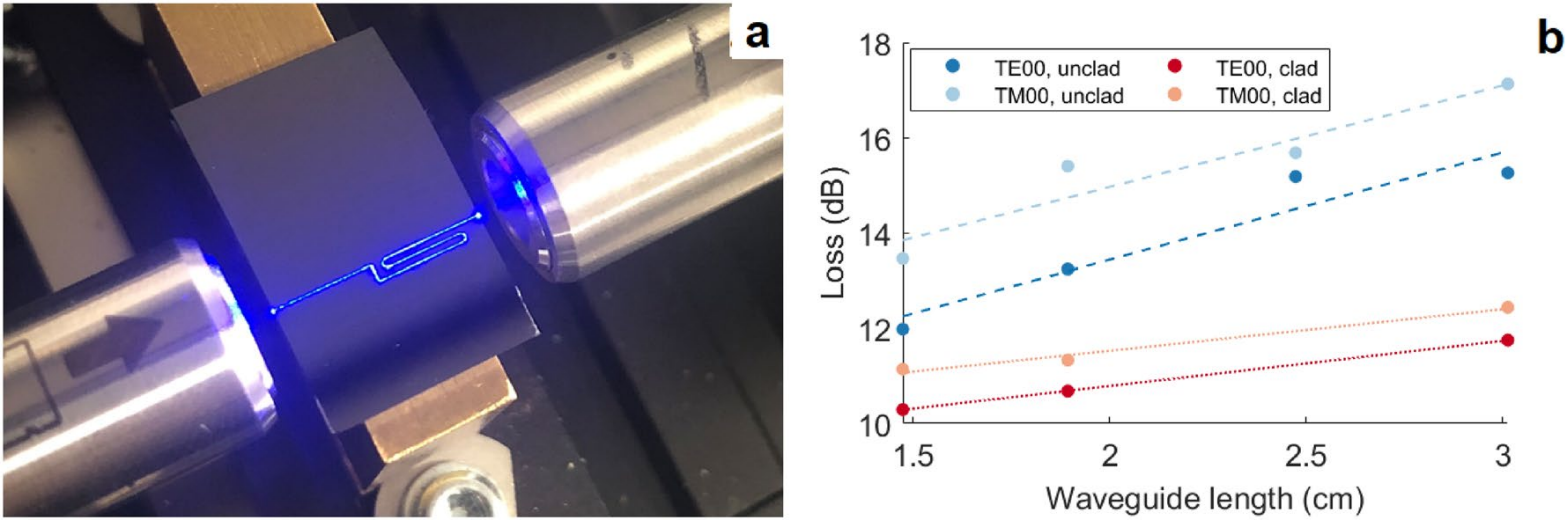
Measurement and observation of propagation losses at 450 nm in an amorphous alumina waveguide without an upper cladding. (**a**) Photograph showing propagation of light along a bent waveguide. Light travels right to left; minimal propagation loss is visible to the naked eye. (**b**) Loss measurements for clad and unclad waveguides of varying lengths.

We fabricated 400 nm-thick waveguides with widths of 700 nm, 850 nm and 1200 nm, providing single-mode operation at 450 nm, 514 nm and 642 nm whilst maintaining a confinement factor higher than 0.7. We characterised waveguide propagation loss using a pseudo-cutback technique: using sets of 4 bent waveguides ranging from 15 to 30 mm in length, we extrapolated propagation loss from the slope of the individual points (Fig. 4). Design, simulation and measurement details are provided in the “Methods” section.

**Figure 5:**
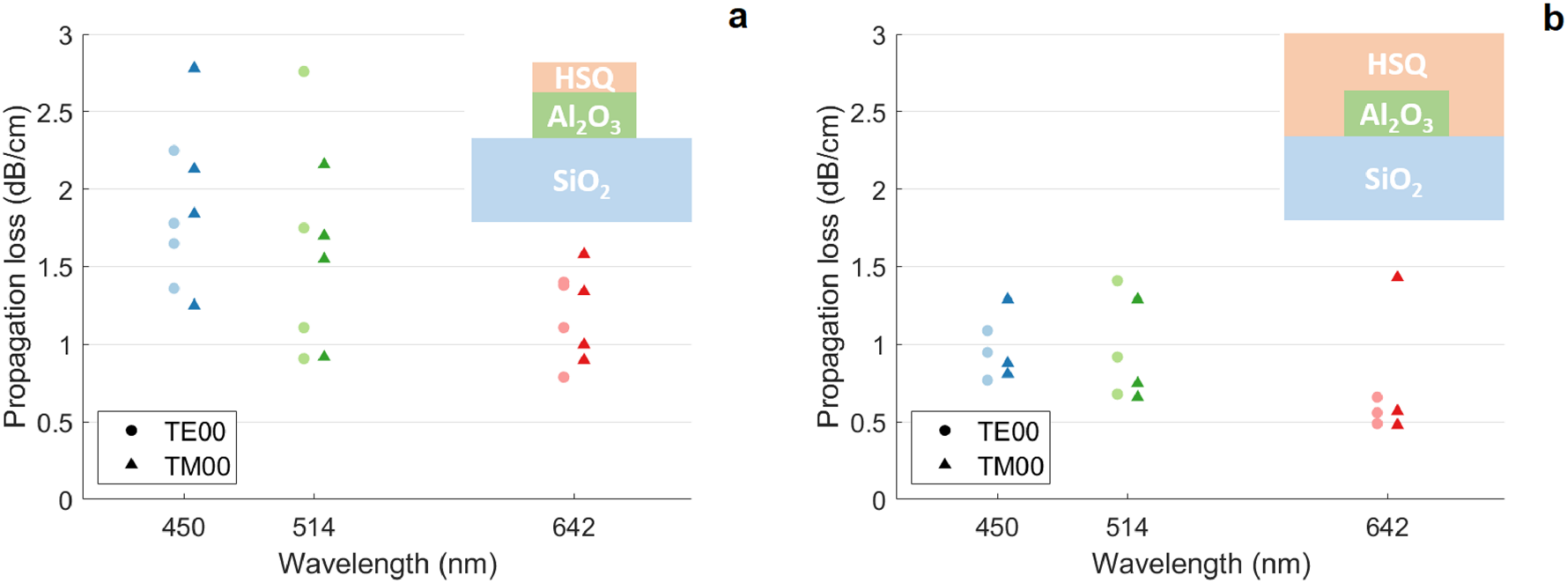
All optical propagation loss results measured in alumina waveguides at 450 nm, 514 nm and 642 nm. (**a**) Waveguides without an upper cladding layer. (**b**) Waveguides with HSQ upper cladding. To aid visualistion, TE00 points have been offset by − 5 nm, TM00 points by + 5 nm. Inset figures show schematics of waveguide cross-sections, not to scale.

We measured losses of four sets of waveguides prior to the application of an upper cladding layer, then applied an HSQ upper cladding to three sets and repeated the measurements. The lowest propagation losses are shown in Table 1, whilst Fig. 5 displays propagation loss results from all waveguides. All loss measurements are provided in the Supplementary Information.

**Table 1:** Lowest attained propagation losses at 450 nm, 514 nm and 642 nm in 400 nm-thick single-mode alumina waveguides.

Wavelength (nm)	Unclad loss (dB/cm)		Clad loss (dB/cm)	
	TE00	TM00	TE00	TM00
642	0.8 (0.78)	0.9 (0.73)	0.6 (0.80)	0.6 (0.78)
514	1.1 (0.88)	0.9 (0.87)	0.7 (0.85)	0.7 (0.85)
450	1.7 (0.92)	1.3 (0.91)	0.8 (0.89)	0.8 (0.89)

The confinement factor for each waveguide mode is provided in brackets.

Given that amorphous alumina typically has a very wide bandgap^10,17^ and the ALD process produces smooth films, we expect loss to be dominated by surface scattering from the waveguide walls, which are well described by the Payne-Lacey model^5^. This model shows that surface scattering loss is mainly related to the roughness of the surface, the refractive index of the materials at the interface, and the wavelength of the propagating mode. Typically, high surface roughness, high index contrast, and short wavelengths result in higher loss.

In line with results from Corato-Zanarella et al.^4^, our AFM measurements of the bottom cladding and alumina core demonstrate negligible surface roughness compared to the typical roughness of an etched sidewall^4,24^, indicating that propagation loss is mainly driven by sidewall roughness. The higher losses prior to cladding application are also consistent with the model because the cladding’s primary effect on losses is in reducing the index contrast at the waveguide walls, which in turn reduces sidewall scattering. Furthermore, while scattering from the top side of the residual mask is already minimal due to reduced interaction of the mode, the application of the cladding results in an index matching at this interface, effectively eliminating any residual loss from the mask surface.

## Discussion

**Figure 6:**
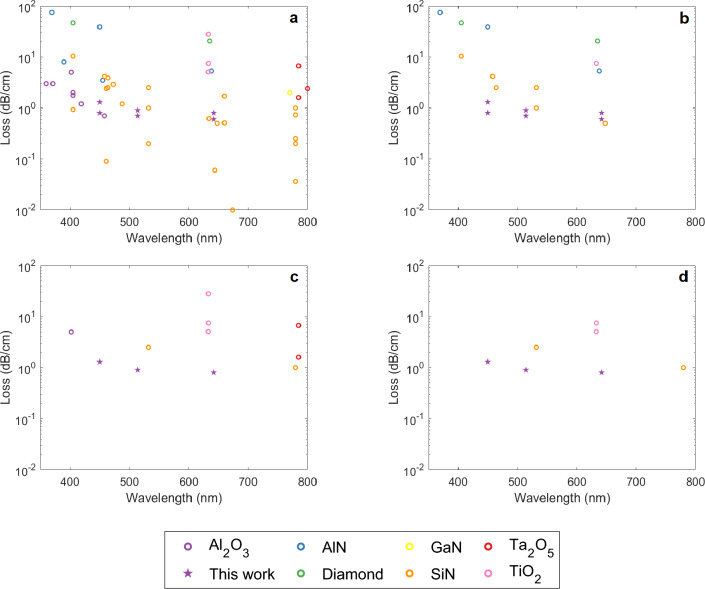
Review of propagation losses achieved in strip/ridge waveguides at wavelengths below 800 nm. Results from this work demonstrate the lowest propagation losses in waveguides with confinement factor > 0.7 at 514 nm and below. The plots show (**a**) All waveguides; (**b**) Single-mode waveguides with confinement factor > 0.7; (**c**) All waveguides without upper cladding; (**d**) Unclad single-mode waveguides with confinement factor > 0.7. Al$_{2}$O$_{3}$:^8,9,21^. AlN:^27–29^. Diamond:^30^. GaN:^31^. SiN:^3,32–41^. Ta$_{2}$O$_{5}$:^42–44^. TiO$_{2}$:^45–47^.

Developing high-quality waveguide definition processes for the visible region is challenging: the high wavelength-dependent losses require material deposition, masking, and etching to be considered concurrently. We selected alumina as a core material given low reported slab^17^ and strip waveguide losses^8,9^, and used ALD to produce a high-quality core layer, with the use of an oxygen plasma for the oxidation half-reaction producing a dramatic reduction in deposition time compared to thermal ALD processes. HSQ provides an etch-resistant, single-step mask with smooth, vertical sidewalls; combined with a highly selective BCl$_{3}$ etch, we demonstrate for the first time the capacity to etch high-confinement alumina waveguides for the visible region.

**Figure 7:**
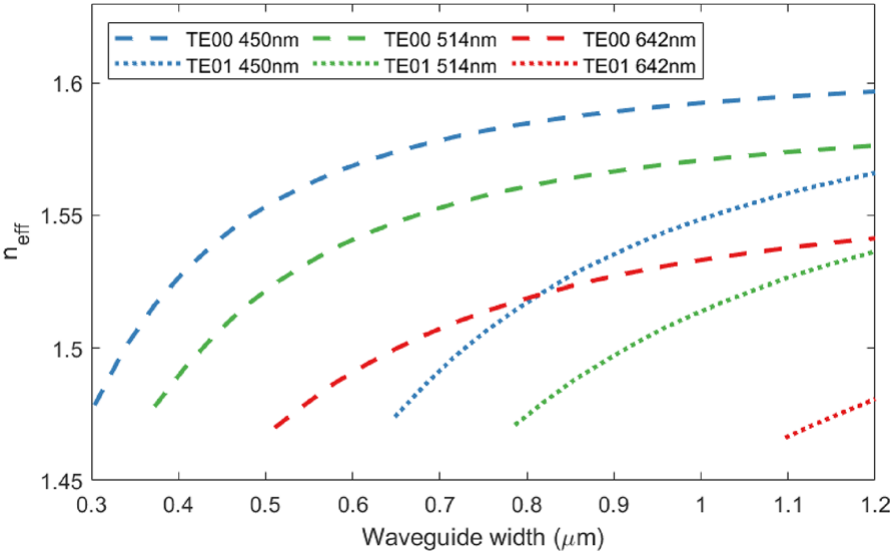
Simulated effective refractive indices ($n_{eff}$) for alumina waveguides without an upper cladding layer. The variation in TE00 and TE01 mode effective indices with waveguide width at 450 nm, 514 nm and 642 nm are shown. Simulations performed using Lumerical MODE finite difference eigenmode (FDE) solver^55^.

Figure 6 provides an overview of published propagation losses in fabricated waveguides, including micro-ring resonators, where the waveguide core layer has been fully etched. This restriction means that several prominent wide-bandgap materials are not listed or are underrepresented in this summary: diamond and lithium niobate, for example, are notably difficult to etch, and whilst rib waveguides are relatively common, fully etched waveguides are not^30,48^. In subfigure (a), it is apparent that alumina and SiN waveguides have demonstrated the lowest losses at blue and UV wavelengths. Limiting this analysis to waveguides with a confinement factor higher than 0.7 (subfigure (b)) discounts a substantial number of results at short wavelengths, including all prior alumina waveguides operating in the visible region; furthermore, it becomes apparent that this work provides the first demonstration of a truly high-confinement low-loss waveguide in the blue, with losses of 0.8 dB/cm and 1.3 dB/cm in clad and unclad waveguides, respectively (Fig. 7). Considering only waveguides without a top cladding (subfigures (c) and (d)), there are very few examples of unclad waveguides with acceptable losses, and even fewer which are also high-confinement; this work demonstrates the first low-loss, unclad high-confinement waveguides in the blue and green.

ALD alumina depositions have demonstrated a low thermo-optic coefficient^3^ and a high damage threshold^49^; combined with the potential for deposition of thick layers with low stress^50^, the material demonstrates potential for use in nonlinear frequency conversion. Nonlinearities can be enhanced by tailoring waveguides’ cross-sectional profile to produce anomalous group velocity dispersion, which typically requires thick waveguides^6,7^. Using our material platform, finite difference eigenmode (FDE) simulations indicate that an unclad waveguide with a core thickness of approximately 800 nm is required to access the anomalous dispersion regime at a pump wavelength of 780 nm. Although the waveguides measured in this work have normal dispersion of about $0.1\,\text {ps}^{2}\text {m}^{-1}$, the fabrication process we present is applicable to the development of thicker waveguides that offer dispersion properties suitable for non-linear applications.

Furthermore, alumina films have demonstrated exceptionally low slab losses at telecoms wavelengths^51^ and have been doped to produce gain media^52,53^. Infrared applications have tended to use substantially thicker films than those used for the visible region, and a sufficiently smooth and selective etch, such as the one described here, could be used to produce extremely low-loss waveguides for the infrared, with integrated gain media.

The use of low-confinement waveguide geometries and upper claddings, where suitable, are practical, loss-minimising choices; however, high-confinement waveguides maximise design flexibility and broaden the scope of what can be achieved within a PIC. The optimisation work required to produce high-confinement waveguides can also be used to improve fabrication processes using thinner films: for example, higher-selectivity etches can be used with thinner masks, improving critical dimension control.

## Methods

### Film characterisation

We measured film thickness using a J. A. Woollam M-2000XI spectroscopic ellipsometer, over a wavelength range of 210 nm to 1700 nm and over an angle range of ${50}^{\circ }$ to ${75}^{\circ }$. We fitted data to a Tauc-Lorentz model^22^, achieving a mean squared error of 12, and extracted optical constants from this fitting.

We measured film roughness using a Bruker Dimension Icon AFM, scanning a $2.5\,{\upmu }\text {m}\times 5\,{\upmu }\text {m}$ area before and after film deposition. We used Gwyddion v.2.59^54^ to generate root mean square roughness values.

### Simulations and design

We used the optical constants derived from ellipsometry to produce a waveguide model in Lumerical MODE R1.2^55^. We used finite difference eigenmode (FDE) simulations to determine allowed transmission modes for various waveguide widths and thicknesses, selected a film thickness for which single-mode operation was feasible for all waveguides (400 nm), calculated confinement factors as the percentage of optical power which propagated within the waveguide core, and selected widths of 700 nm, 850 nm and 1200 nm to maximise confinement whilst allowing single-mode operation at 450 nm, 514 nm and 642 nm. We performed FDE simulations of curved waveguides to establish that bend losses would be negligible at a a bend radius of ${200}\,{\upmu }\text {m}$. We then designed waveguides with a range of propagation lengths from 15 to 30 mm; each waveguide has the same number of bends, so that any unanticipated bending loss is the same in all waveguides and any variation between results is primarily from differences in propagation loss.

### Fabrication

We deposited an amorphous alumina film on an Si wafer with a pre-grown, $5\;{\upmu }\text {m}$-thick thermal SiO$_{2}$ layer. We used an Oxford Instruments FlexAL ALD system to perform a plasma ALD process (${200} \; ^{\circ }\text {C}$; TMA and O$_{2}$ plasma, 400 W) to deposit 400 nm of alumina. We used a 700 nm-thick HSQ mask (Dow FOx-16; spin 2000 rpm, 60 s; softbake 15 min $92\; ^{\circ }\text {C}$), exposed using a 100 kV Raith PG5200 EBL system (exposure dose $1450\,{\upmu }\text {C}\,\text {cm}^{-2}$ over 4 passes; beam current 2 nA; beam step size 1 nm; field boundary overlap $1\,{\upmu }\text {m}$; sample height offset $1\,{\upmu }\text {m}$). After a post-exposure bake (hotplate; 60 s; $180\,^{\circ }\text {C}$) we developed samples in 25% tetramethylammonium hydroxide (TMAH) then used an Oxford Instruments Plasmalab 180 ICP system for pattern transfer (BCl$_{3}$; ICP power 600 W; platen power 25 W). We determined resist etch rate by ellipsometric measurement of a monitor sample before and after etch, and alumina etch rate by measuring waveguide step height with a stylus profiler before and after etch. We cleaved facets for end-fire coupling using a manual scribe and break process. Following testing in their unclad state, we applied an upper cladding: a 700 nm-thick layer of HSQ (Dow FOx-16; spin 2000 rpm, 60 s; softbake 15 min $92\;^{\circ }\text {C}$; convection oven 72 h $180\;^{\circ }\text {C}$).

### Loss testing

We measured propagation loss using a pseudo-cutback method: for each waveguide width, we fabricated bent waveguides with lengths from 15 to 30 mm on a single chip, to minimise differences in optical facet quality. The optical test setup uses pigtailed diode lasers (450, 514, and 642 nm), each of which can be attached to a fibre collimator, producing a free-space beam; a half-wave plate and a Glan-laser prism in the beam path control beam polarisation and attenuation. An aspheric lens focuses the beam on the optical facet, and a second aspheric lens aligned to the output facet focuses the beam on a Si photodiode.

### Supplementary Information

Supplementary Information.

## Data Availability

Upon acceptance, loss measurement data will be stored in an institutional repository and a DOI will be issued.
